# Endoscopic submucosal dissection for a squamous cell carcinoma invading the lamina propria in the floor of the mouth

**DOI:** 10.1055/a-2599-7610

**Published:** 2025-06-26

**Authors:** Yueming Zhang, Shibo Song, Lizhou Dou, Qingmiao Zhao, Guiqi Wang, Shun He

**Affiliations:** 1 Department of Endoscopy, National Cancer Center/National Clinical Research Center for Cancer/Cancer Hospital, Chinese Academy of Medical Sciences and Peking Union Medical College, Beijing, China; 2 26447 Endoscopy Center, Peking University First Hospital, Beijing, China

Oral cancer globally affects 389.485 people annually, with about 5% occurring in the floor of the mouth (FOM) 1 2 . Surgical resection remains the primary treatment for oral squamous cell carcinoma, but it can greatly affect the patient’s quality of life 3 . For the first time, we present a case of superficial oral cancer in the FOM that was safely and successfully cured using endoscopic submucosal dissection (ESD).

A 58-year-old man was diagnosed with high grade intraepithelial neoplasia (HGIN) in the FOM during follow-up after esophageal ESD ( Fig. 1 ). Contrast-enhanced computed tomography showed no cervical lymph node metastasis. Considering the impact of surgery on quality of life and the malignant potential of HGIN, the patient underwent ESD. The lesion was removed en bloc following ESD protocols, including marking, submucosal injection, submucosal dissection, and electrocoagulation hemostasis ( Fig. 2 , Fig. 3 , Video 1 ). Lidocaine was not used in the submucosal injection. The procedure lasted 40 minutes.

**Fig. 1:**
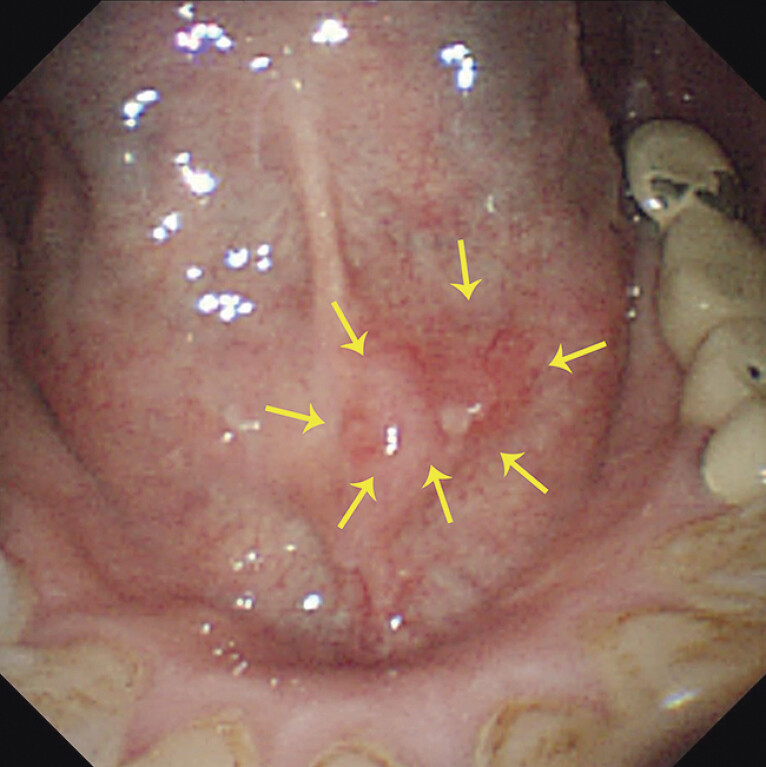
A superficial oral lesion in the floor of the mouth (a circle of yellow arrows surrounds the lesion).

**Fig. 2:**
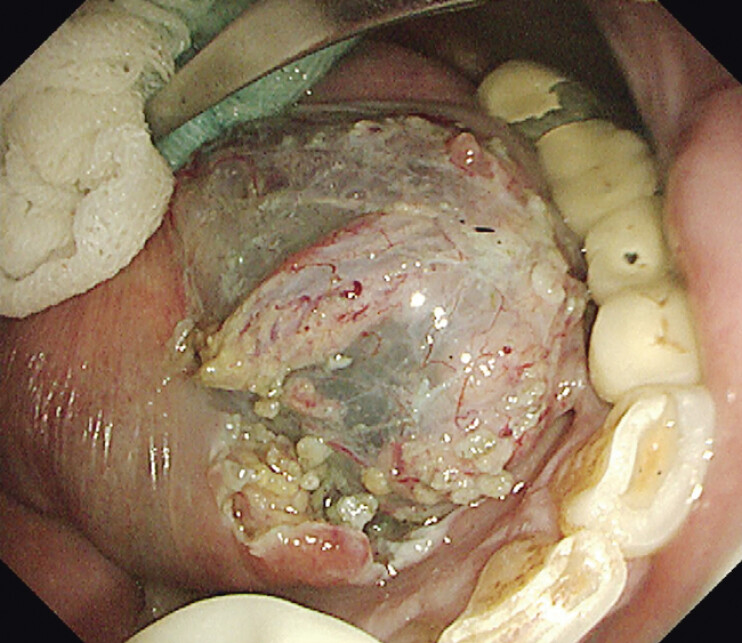
A defect in the floor of the mouth after endoscopic submucosal dissection.

**Fig. 3:**
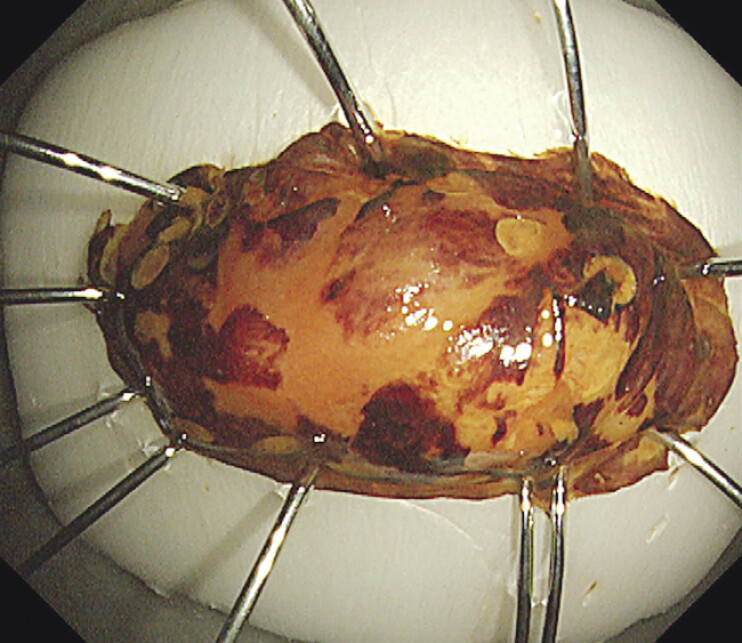
The specimen removed en bloc and stained with iodine.

A superficial oral lesion in the floor of the mouth was completely removed via endoscopic submucosal dissection.Video 1

On postoperative day (POD) 2, enteral feeding was initiated via a nasogastric tube, and intermittent mouth rinses with chlorhexidine mouthwash or lidocaine gel were performed for pain control. After the nasogastric tube was removed on POD 5, the patient transitioned to a liquid diet and was discharged on POD 6 without adverse events. Histology confirmed complete excision of squamous cell carcinoma invading the lamina propria, with margins approximately 2000 μm from the horizontal edge and 1100 μm from the basal edge ( Fig. 4 ). The 3-month follow-up nasopharyngoscopy showed good healing and no recurrence ( Fig. 5 ).

**Fig. 4:**
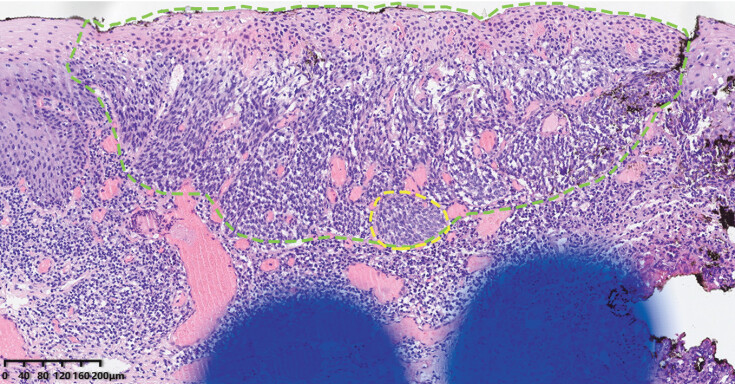
Histopathological image of the lesion (the green dashed line outlines the extent of high grade intraepithelial neoplasia; the yellow dashed line outlines a small focus of squamous cell carcinoma invading the lamina propria).

**Fig. 5:**
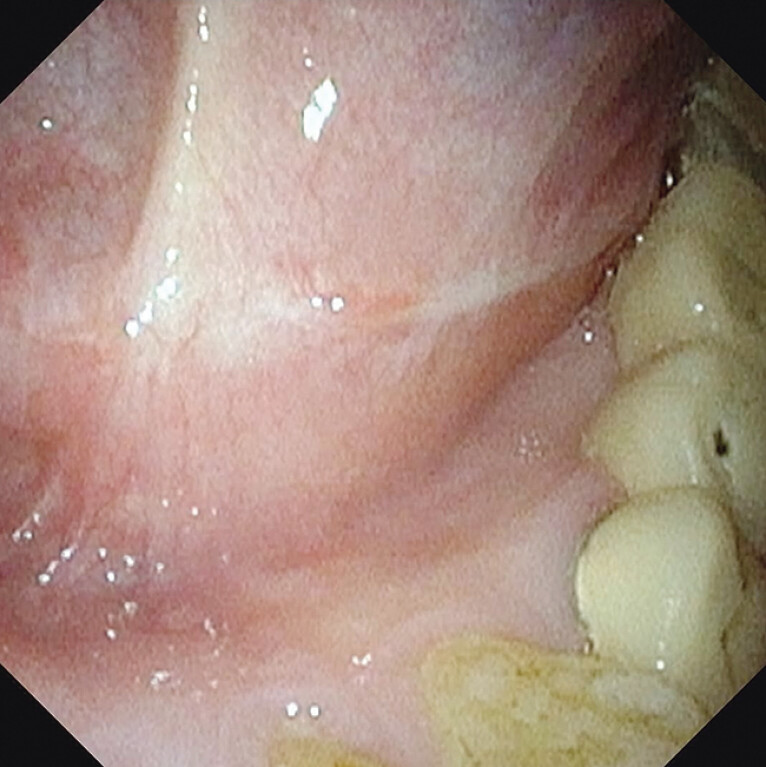
The defect on postoperative month 3.

ESD has not been previously reported for superficial oral cancer in the FOM. In this case, the lesion was completely removed via ESD without impairing the patient’s oral appearance or function. During ESD, protecting the deep lingual vessels and nerve is crucial, as is the assistant’s role in exposure due to the unique location. Further accumulation of clinical experience is warranted.

Endoscopy_UCTN_Code_TTT_1AO_2AG_3AD

## Notes

Endoscopy E-Videos https://eref.thieme.de/e-videos
*E-Videos* is an open access online section of the journal *Endoscopy* , reporting on interesting cases and new techniques in gastroenterological endoscopy. All papers include a high-quality video and are published with a Creative Commons CC-BY license. Endoscopy E-Videos qualify for HINARI discounts and waivers and eligibility is automatically checked during the submission process. We grant 100% waivers to articles whose corresponding authors are based in Group A countries and 50% waivers to those who are based in Group B countries as classified by Research4Life (see: https://www.research4life.org/access/eligibility/ ). This section has its own submission website at https://mc.manuscriptcentral.com/e-videos .

## References

[1] Bray F Laversanne M Sung H Global cancer statistics 2022: GLOBOCAN estimates of incidence and mortality worldwide for 36 cancers in 185 countries CA Cancer J Clin 2024 74 229 263 10.3322/caac.21834 38572751

[2] Joo YH Cho JK Koo BS Guidelines for the surgical management of oral cancer: Korean Society of Thyroid–Head and Neck Surgery Clin Exp Otorhinolaryngol 2019 12 107 144 10.21053/ceo.2018.01816 30703871 PMC6453784

[3] Pfister DG Spencer S Adelstein D Head and neck cancers, version 2.2020, NCCN clinical practice guidelines in oncology J Natl Compr Canc Netw 2020 18 873 898 10.6004/jnccn.2020.0031 32634781

